# German Society of Biomechanics (DGfB) Young Investigator Award 2019: Proof-of-Concept of a Novel Knee Joint Simulator Allowing Rapid Motions at Physiological Muscle and Ground Reaction Forces

**DOI:** 10.3389/fbioe.2019.00244

**Published:** 2019-09-27

**Authors:** Florian Schall, Andreas M. Seitz, Steffen Hacker, Stefan van Drongelen, Sebastian I. Wolf, Anita Ignatius, Lutz Dürselen

**Affiliations:** ^1^Institute of Orthopaedic Research and Biomechanics, Centre of Trauma Research, Medical Centre, Ulm University, Ulm, Germany; ^2^Clinic for Orthopaedics and Trauma Surgery, Heidelberg University Hospital, Heidelberg, Germany; ^3^Dr. Rolf M. Schwiete Research Unit for Osteoarthritis, Orthopaedic University Hospital Friedrichsheim GmbH, Frankfurt/Main, Germany

**Keywords:** knee, biomechanics, simulator, muscle forces, *in vitro*, contact pressure, rapid movement

## Abstract

The *in vitro* determination of realistic loads acting in knee ligaments, articular cartilage, menisci and their attachments during daily activities require the creation of physiological muscle forces, ground reaction force and unconstrained kinematics. However, no *in vitro* test setup is currently available that is able to simulate such physiological loads during squatting and jump landing exercises. Therefore, a novel knee joint simulator allowing such physiological loads in combination with realistic, rapid movements is presented. To gain realistic joint positions and muscle forces serving as input parameters for the simulator, a combined *in vivo* motion analysis and inverse dynamics (MAID) study was undertaken with 11 volunteers performing squatting and jump landing exercises. Subsequently, an *in vitro* study using nine human knee joint specimens was conducted to prove the functionality of the simulator. To do so, slow squatting without muscle force simulation representing quasi-static loading conditions and slow squatting and jump landing with physiological muscle force simulation were carried out. During all tests ground reaction force, tibiofemoral contact pressure, and tibial rotation characteristics were simultaneously recorded. The simulated muscle forces obtained were in good correlation (0.48 ≤ R ≤ 0.92) with those from the *in vivo* MAID study. The resulting vertical ground reaction force showed a correlation of R = 0.93. On the basis of the target parameters of ground reaction force, tibiofemoral contact pressure and tibial rotation, it could be concluded that the knee joint load was loaded physiologically. Therefore, this is the first *in vitro* knee joint simulator allowing squatting and jump landing exercises in combination with physiological muscle forces that finally result in realistic ground reaction forces and physiological joint loading conditions.

## Introduction

For biomechanical *in vitro* investigations of human knee joint specimens, different types of knee joint simulators have been introduced. The complexity of the human knee joint requires a sophisticated design of such simulators. Fundamentally, a distinction can be made between horizontal knee joint simulators, vertical simulators, so-called Oxford-Rigs, and simulators driven by a robotic arm.

Horizontal knee joint simulators are particularly characterised by the horizontal position of the knee joint specimens (Blankevoort et al., 1988; Hirokawa et al., 1991; Torzilli et al., 1994; Bach and Hull, 1995; Dürselen et al., 1995; Omori et al., 1997; Ahmad et al., 1998; Kiguchi et al., 1999; Stukenborg-Colsman et al., 2002b; Hofer et al., 2011). Typically, the femur or the tibia is fixed to the simulator base or to a moveable swing arm, which is responsible for the flexion and extension movements, whereas the opposite side provides all necessary degrees of freedom (Heinrichs et al., 2017). Robotic arm systems (Rudy et al., 1996; Livesay et al., 1997; Li et al., 1999; Lo et al., 2008; Diermann et al., 2009; Goldsmith et al., 2013) are comparable to horizontal simulators, but the knee joint is moved along a previously determined passive motion path in which all external forces and moments acting on the knee joint are minimal (Lorenz et al., 2013). The Oxford-Rig (Kumagai et al., 2002; Lo et al., 2008) is characterized by an upright and vertical fixation of the knee joint as well as a hip- and an ankle-joint assembly (Zavatsky, 1997). The hip-joint assembly can be moved vertically, thereby providing flexion and extension to the knee joint. As a variation of the Oxford-Rig design, there are impact simulators mimicking impacts on the knee joint using falling weights (Withrow et al., 2006; Kiapour et al., 2016).

Some of these simulators are able to mimic muscle forces acting across the knee joint. This is realised by weights or actuators and steel cables, which are connected to the bone at the anatomical insertion sites or directly to the muscles by special clamps. Typically, the quadriceps muscle, the two-headed gastrocnemius muscle or the hamstring muscles are simulated (Hirokawa et al., 1991; Shoemaker et al., 1993; Bach and Hull, 1995; Dürselen et al., 1995; Ahmad et al., 1998; Li et al., 2002; Gill et al., 2003; Hofer et al., 2011; Heinrichs et al., 2017). However, in most cases the applied muscle forces are relatively low and attain only values of up to 200 N (e.g., simulating the quadriceps muscle; Dürselen et al., 1995; Withrow et al., 2006). This means that neither physiological loading conditions inside the knee joint nor a physiological ground reaction force (generated by muscle forces) can be achieved. Furthermore, adapting muscle forces over time or with a changing knee flexion angle is rarely possible with current knee joint simulators, resulting in only slow knee joint movements (Stukenborg-Colsman et al., 2002a).

The most common methods for creating knee joint movement in current knee simulators are either passive knee flexion or control of the knee flexion angle or ground reaction force via a muscle force control-loop (Stukenborg-Colsman et al., 2002a; Victor et al., 2009). However, because the different knee-spanning muscles influence each other, leading to a statically indeterminate mechanical system, a real-time control of several simultaneously acting muscles is difficult to accomplish for dynamic movements, for example, drop jumping. Consequently, such control-loop mechanisms realised in current simulators only allow slow joint motions with flexion-extension rates as low as ~1°/s (Churchill et al., 1998; Lo et al., 2008), corresponding to quasi-static testing conditions. Some of the existing Oxford-Rig-like knee joint simulators are able to simulate almost physiological ground reaction forces or body weights (Elias et al., 2002; Maletsky and Hillberry, 2005). These simulators are able to simulate movements of up to 12°/s, which is still much lower than those required for jump landing (145°/s).

To actually be able to achieve realistic *in vitro* testing conditions as they occur during daily activities, knee joint movements and muscle forces resulting in physiological joint and ground reaction forces are required. Therefore, the aim of this study was to develop a novel knee joint simulator for *in vitro* testing of squatting and drop jump movements at realistic speeds and joint forces.

## Materials and Methods

### Technical Description

**Figure 1:**
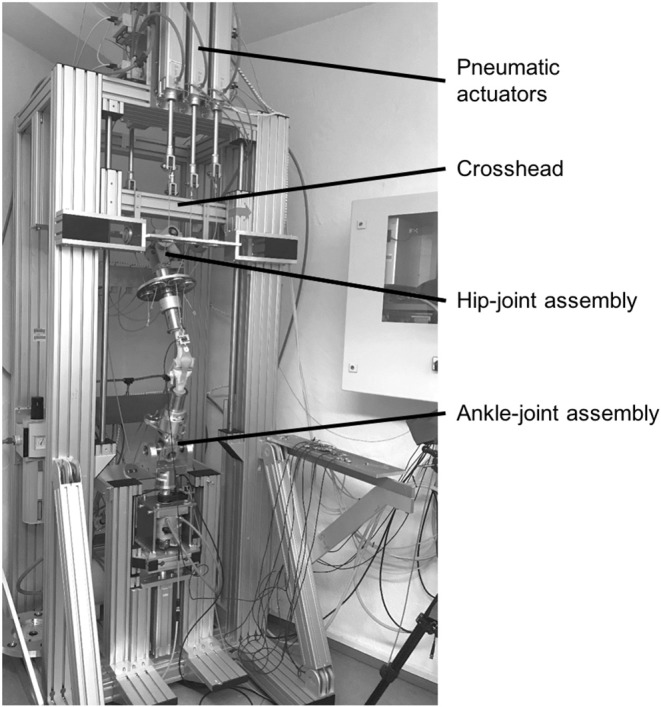
Knee joint simulator with a knee joint model fixed between the hip- and ankle-joint assemblies, crosshead for vertical hip displacement and pneumatic actuators for muscle force simulation.

The mechanical construction of the novel knee joint simulator, which is based on the design of an Oxford-Rig (Bourne et al., 1978), comprises a base frame, a hip-joint assembly and an ankle-joint assembly (Figure 1). The hip-joint assembly consists of a universal joint. It provides three degrees of freedom, including flexion/extension, abduction/adduction, and vertical linear displacement. Vertical displacement is achieved by the hip-joint assembly attached to a crosshead, which can be moved vertically along a guided ball bearing driven by an electrical servomotor (EMMS-AS-140-L-HS-RMB, Festo AG & Co. KG, Esslingen, Germany) with a linear axis at a maximum crosshead speed of 670 mm/s, corresponding to a maximum angular velocity of 350°/s. It must be noted that the simulation of hip movement results only in knee flexion and extension without creating any joint or ground reaction forces. The ankle-joint assembly has two degrees of freedom, allowing flexion/extension, and abduction/adduction. Additionally, because of an additional bearing, the tibia is free to rotate internally and externally. Consequently, the knee joint simulator allows unconstrained movement in 6° of freedom (Zavatsky, 1997).

The nine most relevant knee-spanning muscles are simulated to achieve physiological loading conditions and ground reaction forces. These muscles are the Musculus (M.) vastus medialis, M. vastus lateralis, M. vastus intermedius, M. rectus femoris, M biceps femoris, M. semitendinosus, M. semimembranosus, M. gastrocnemius medialis and M. gastrocnemius lateralis. Because of similar anatomical tensile directions, the M. vastus intermedius and M. rectus femoris as well as the M. semitendinosus and M. semimembranosus are combined and simulated as a single-acting muscle, respectively. In total, seven pneumatic actuators (DNCI-63-300-P-A, Festo AG & Co. KG) are used for muscle force simulation, which are located in the upper and lower areas of the base frame. Bicortical screws are positioned at the anatomical insertion sites of the respective muscles. Steel cables connect the pneumatic actuators and the bicortical screws to allow for muscle force simulation. Seven uniaxial force sensors (KD40S, ME-Messsysteme GmbH, Henningsdorf, Germany) are built in the steel cables to measure the applied muscle forces, respectively. Additionally, a linear pneumatic actuator and a rotational pneumatic actuator are located below the ankle-joint assembly to simulate axial shock loads and tibial distortion moments, respectively. To measure the ground reaction forces and moments, a six-axis force/torque sensor (K6D68, ME-Messsysteme GmbH) is fixed directly below the ankle-joint assembly. Therefore, the hip movement creates the knee joint flexion and extension, while the seven pneumatic actuators are used to simulate muscle forces that result in the related ground reaction forces. That is, without the muscle force simulation there would be no resultant ground reaction force, but just knee joint flexion and extension.

**Figure 2:**
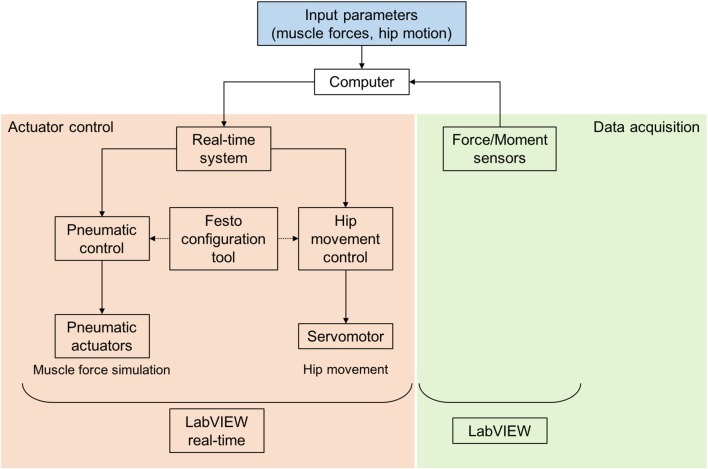
Control of the dynamic knee joint simulator with a real-time system, Festo configuration tool for parameterisation, control of the pneumatic and electrical actuators, registration of force sensors and LabVIEW and LabVIEW real-time applications.

The simulator is designed to work in a combination of position-control and force-control modes (Figure 2). The position-controlled, linear displacement of the hip is directly linked to the flexion and extension of the knee joint. The muscle forces are applied in a force-controlled mode. For these purposes, both the linear displacement of the hip joint as a function over time and the muscle forces as a function over time serve as input parameters for the knee joint simulator. These input values were obtained from a combined motion analysis and inverse dynamic (MAID) study on 11 healthy volunteers performed in the motion laboratory of a cooperation partner (Clinic for Orthopaedics and Trauma Surgery, Heidelberg University Hospital, Heidelberg, Germany). In doing so, the kinematics and kinetics of the subjects were measured. These values together with the anthropometric data were used to calculate the acting muscle forces by means of an inverse dynamic musculoskeletal simulation.

The input parameters for muscle forces and hip position derived from the MAID study were assigned to the respective actuators (parameterisation) by using a pneumatic configuration tool (Festo AG & Co. KG) (Figure 2). The simultaneous control of all actuators is realised utilising a real-time system (cRIO-9064, National Instruments, Austin, TX, USA) and a custom-made software application (LabVIEW 2014, National Instruments). The data acquisition of the uniaxial muscle force sensors and the six-axis ground reaction force/torque sensor is achieved by another custom-made LabVIEW application (National Instruments). Both applications enable rapid and real-time control, signal processing and data acquisition.

### Combined Motion Analysis and Inverse Dynamic Study (MAID)

Eleven healthy adults (six women, five men, age = 30.9 ± 9.3 a, weight = 71.8 ± 17.1 kg, height = 1.77 ± 0.11 m) were examined in a subject study (IRB approval no. S-081/2015 Heidelberg University). Three-dimensional (3D) motion analysis was performed with a 12-camera optoelectronic system (Vicon Motion Systems Ltd., Oxford, England) operating at 120 Hz. The marker protocol used for this study was the Plugin-Gait lower body marker set (Vicon Motion Systems, Oxford, UK) with additional markers on the subject's thorax (spinous process of the 7th cervical vertebrae, left and right acromion, and incisura jugularis) as well as on the medial malleolus and the medial femoral condyles. Additionally, two force-measuring platforms (Kistler Instruments AG, Winterthur, Switzerland) were used to synchronously collect kinetic data at 1,080 Hz. Joint kinematics and joint kinetics were obtained using the inverse dynamics equations approach with the software Plugin-Gait (Vicon Nexus 2.0, Vicon Motion Systems, Oxford, UK) following Kadaba et al. and Davis et al. The subjects performed slow squats from 0° to 70° knee flexion and a double-legged jump landing from a height of 30 cm to generate different datasets. Consequently, the movements and positions of the hip, knee, and ankle joint with the resulting flexion angles and the ground reaction forces were determined. These data were used to calculate the muscle forces acting across the knee joint over time using a generic full-body musculoskeletal model to analyse the motion data in OpenSim 3.3 (Delp et al., 2007). The metatarsophalangeal and subtalar joints were fixed in anatomically neutral positions for all analyses, as has been done recently by other authors (O'Connor et al., 2018). A fourth order zero-lag low-pass filter with a cut-off frequency of 10 Hz was applied to ground reaction forces, whereas a Woltring filter with MSE 10 was used to smooth the kinematical data (Woltring, 1991). Input for the model was created using custom MATLAB routines (2014b, The MathWorks, Inc., Natick, MA, USA), based on MATLAB scripts for processing data from the simtk.org website. The model was scaled to the dimensions of each subject based on a static trial. Inverse kinematics and inverse dynamics were performed to calculate joint angles and joint moments. Muscle forces were calculated using static optimisation.

### *In-vitro* Study

**Figure 3:**
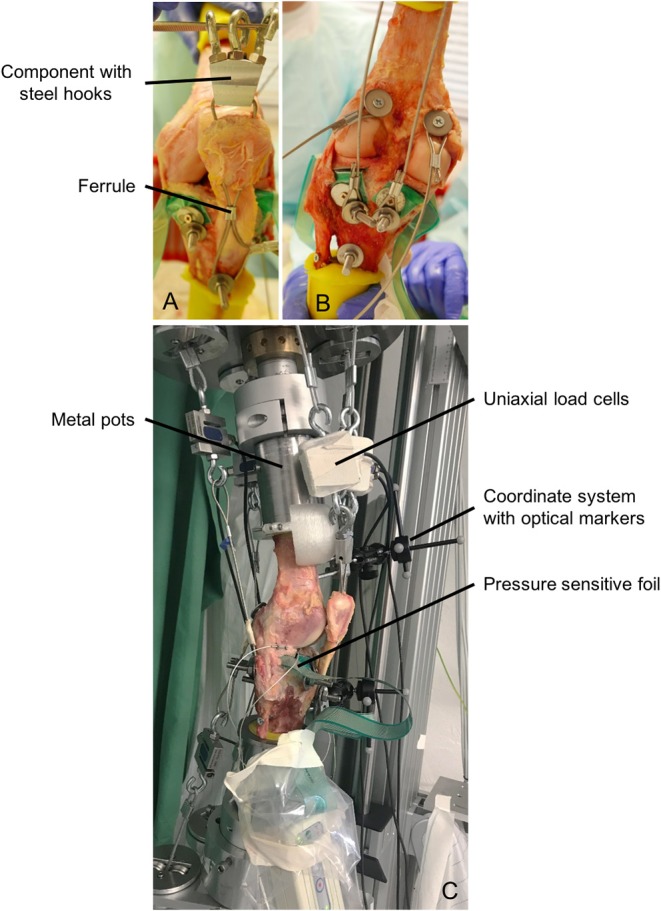
**(A)** Muscle force simulation of the quadriceps muscle using a threaded rod, steel cable, component with steel hooks and a ferrule. **(B)** Muscle force simulation of the hamstring and gastrocnemius muscles using threaded rods, dowels and steel cables. **(C)** Specimen fixed in the dynamic knee joint simulator with cylindrical metal pots, uniaxial load cells for measuring muscle forces, pressure-sensitive foil for measuring the tibiofemoral contact pressure and coordinate systems with optical markers for measuring the kinematics.

After thawing overnight, the skin and muscles of nine human cadaveric knee joint specimens (age: 61.5 ± 5.5 years, body weight: 62.3 ± 7.2 kg, body mass index: 21.2 ± 1.0, Science Care, Inc. Phoenix, AZ, USA; IRB approval no. 300/12, Ulm University) were completely removed and the femur and tibia were exposed. The proximal fibula was fixed to the tibia using a cortical screw and resected 2 cm below the fibula head. The femur and tibia were cut at a distance of 12 cm to the knee gap and moulded in metal pots using polymethylmethacrylate (Technovit 4000, Kulzer GmbH, Wehrheim, Germany) (Figure 3C). The joint capsule was carefully opened, the patella was exposed and the infrapatellar fat resected. The coronary meniscal attachments were incised anteriorly and posteriorly to allow the insertion of a pressure-sensitive Tekscan foil (I-Scan system (Type 4000), Tekscan Inc., Boston, MA, USA) on the tibial plateau to measure mean and peak tibiofemoral contact pressure. The pressure sensor was anteriorly and posteriorly secured to the tibia with a screw to minimise motion of the sensor during testing.

Because of large muscle forces of up to 1,000 N acting on the knee joint specimens, a rigid fixation of steel cables at the anatomical insertion sites is necessary. Therefore, to simulate the anterior thigh muscles, a hole was drilled through the insertion site of the patellar tendon at the tibial tuberosity and a threaded rod was inserted and secured using a locknut. Furthermore, two holes were drilled through the patella and a steel cable was attached to the threaded rod and passed through these two holes. To ensure a guidance of the patella during movement, a ferrule (Carl Stahl Technocables GmbH, Süßen, Germany) was mounted below the patella inside the steel cables (Figure 3A). Above the patella, a component with three bolted steel hooks was fixed to establish the connection between the anatomical insertion site and the uniaxial load cells and the pneumatic actuators. The simulation of the hamstring muscles was also performed using threaded rods in the anatomical insertion sites of the muscles (Figure 3B). The M. biceps femoris inserts at the fibula head, the M. semitendinosus inserts at the pes anserinus at the medial tibial tuberosity and the semimembranosus muscles inserts at the medial tibial condyle. For the simulation of the calf muscles (M. gastrocnemius medialis, M. gastrocnemius lateralis), dowels were used, which were attached in the points of origin at the medial and lateral femoral condyles (Figure 3B). All steel cables were additionally guided by self-aligning pivoting units to ensure the best possible anatomical line of action. Throughout the entire preparation process and all tests, the knee joint specimens were kept moist with saline solution.

After preparation, the knee joint specimens were fixed in an upright position in the knee joint simulator using cylindrical metal pots (Figure 3C). Furthermore, the pneumatic actuators were connected to the steel cables and the uniaxial force sensors (Figure 3C). In a first step and to precondition the knee joint specimen, a slow squat without any muscle force simulation was performed. The knee joint specimen was flexed from 10° knee flexion to 70° and extended back to 10° at a flexion velocity of 5°/s. This motion was repeated with muscle force simulation according to the target muscle forces obtained from the MAID study. Finally, we simulated a jump landing movement with muscle force simulation during which the specimen was flexed from 10° to 50° at a velocity of ~180°/s and extended back from 50° to 10° at a velocity of ~120°/s (see Supplementary Video). The hip acceleration and deceleration during flexion was set to 2.5 m/s^2^ and during extension to 1.5 m/s^2^. The jump landing performed by the subjects (MAID study) lasted 420 ms.

Before starting the jump landing simulation, preload muscle forces between 50 and 300 N were applied to stabilise the knee joint. During slow squat movement and jump landing, tibiofemoral contact pressure was continuously recorded (K-Scan™, Tekscan Inc.). The knee joint kinematics were recorded utilising a marker-based 3D-camera system (Optitrack, NaturalPoint, Inc., OR, USA). During jump landing, the ground reaction force and the applied muscle forces were additionally recorded at a sampling rate of 1 kHz using custom-made LabVIEW software (National Instruments).

### Statistical Analysis

The Bravais-Pearson correlation coefficient (R) was used to compare the actual and target values (MAID) of the ground reaction force and the applied muscle forces during jump landing. Values >0.5 of the multiple correlation coefficients show a moderate relationship and values >0.8 show a strong linear relationship. Gaussian distribution of the tibiofemoral peak and mean pressure distribution data using the Shapiro-Wilk test (Shapiro and Wilk, 1965), resulted in normally distributed data. Therefore, a One-Way ANOVA with a *post-hoc* LSD test were performed to compare mean and peak tibiofemoral contact pressure between slow squat with and without muscle force simulation and a drop jump landing with muscle force simulation of the lateral and the medial compartment, respectively. Differences in medial and lateral tibiofemoral contact pressure were investigated using a paired Student's *t*-test. A statistics software package (SPSS V24. IBM Corp., Armonk, USA) was used to conduct the statistical analyses, while a *p*-value < 0.05 was considered significant and standard Bonferroni correction applied where necessary.

## Results

### Muscle Forces

**Figure 4:**
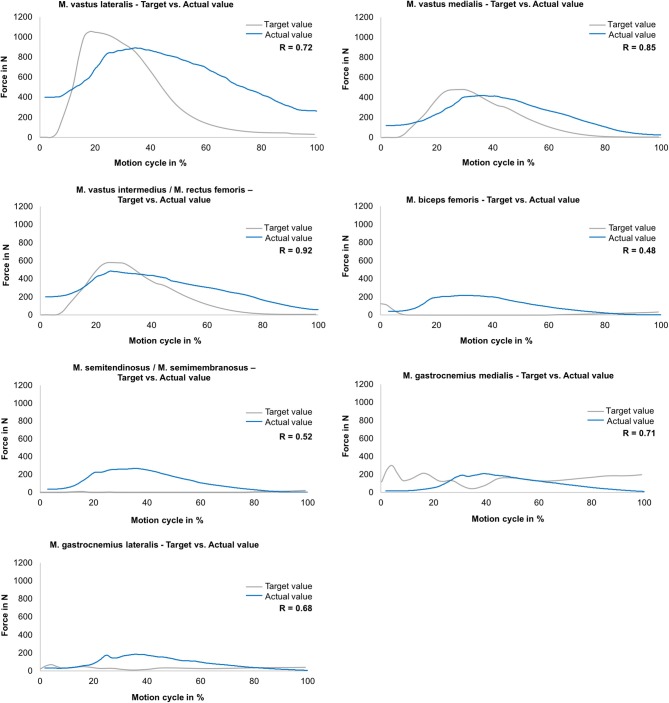
Muscle force simulation—comparison between the actual (mean values) and target muscle forces (gained in MAID study) as a function of the motion cycle (duration: 540 ms) during jump landing for M. vastus lateralis, M. vastus medialis, M. vastus intermedius/M. rectus femoris, M. biceps femoris, M. semitendinosus/M. semimembranosus, M. gastrocnemius medialis and M. gastrocnemius lateralis (*n* = 9).

All simulated mean actual muscle forces and target muscle forces gained from the MAID study are presented as a function of the motion cycle for the jump landing movement (Figure 4). The target force of the M. vastus lateralis increased within 80 ms to a maximum value of 1,050 N. The simulated muscle force was ~15% lower with a delay of ~60 ms, leading to a correlation of R = 0.72. The target force of the M. vastus medialis increased within 120 ms to 480 N, whereas the simulated muscle force was ~10% lower with a delay of 60 ms (R = 0.85). The target force of the M. vastus intermedius and M. rectus femoris muscles increased to 580 N within 100 ms. The simulation of this muscle group was ~12% lower with a delay of 40 ms (R = 0.92). The target values of the hamstring muscles (M. biceps femoris, M. semitendinosus/M. semimembranosus) and of the gastrocnemius muscles (M. gastrocnemius medialis, M. gastrocnemius lateralis) were between 0 and 200 N, leading to correlations of R = 0.48, R = 0.52, R = 0.71 and R = 0.68, respectively.

### Ground Reaction Force

**Figure 5:**
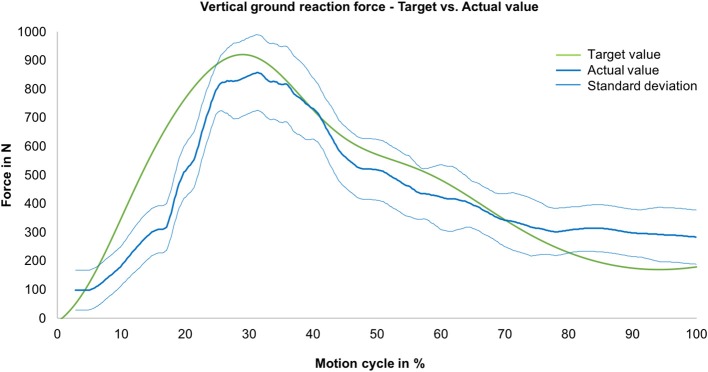
Vertical ground reaction force—comparison between the actual (mean value, blue line) with standard deviation (envelope curve, light blue lines) and target ground reaction forces (measured during MAID study, green line) as a function of the motion cycle (duration: 540 ms) (*n* = 9).

Regarding the ground reaction force in the vertical direction during jump landing, a strong correlation (R = 0.93) between the mean actual value and the target value was determined (Figure 5). At the beginning of the movement, the vertical ground reaction force reached values of ~100 N because of the previously described offset muscle forces. In further progression, forces of up to 860 N were generated.

### Knee Contact Pressure

**Figure 6:**
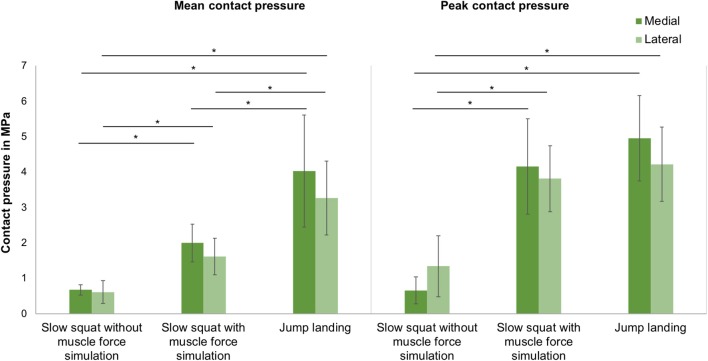
Mean and peak contact pressure (mean ± SD) in the medial and lateral compartments for slow squat without muscle force simulation, slow squat with muscle force simulation and a jump landing exercise. ^*^p ≤ 0.05 (*n* = 9).

Mean and peak contact pressure data for a slow squat without and with muscle force simulation and for a jump landing motion for the medial and lateral knee compartments are presented in Figure 6, respectively. The One-way ANOVA indicated a significant difference (*p* < 0.001) for all mean and peak pressure measurements. LSD *post-hoc* testing revealed a significant increase in the mean and peak contact pressures in the medial and lateral compartments between a slow squat without muscle force simulation and both slow squat with muscle force simulation (*p* < 0.04) and jump landing with muscle force simulation (*p* < 0.001). Peak contact pressures were not different (*p* > 0.187) when comparing the slow squat with muscle force simulation and the jump landing with muscle force simulation. Mean contact pressure calculations indicated significantly higher values for the jump landing (*p* < 0.001) compared to the slow squatting with muscle force simulation. Comparisons of the medial and lateral peak and mean contact pressure indicated no difference (*p* > 0.067) between the compartments.

### Kinematics

**Figure 7:**
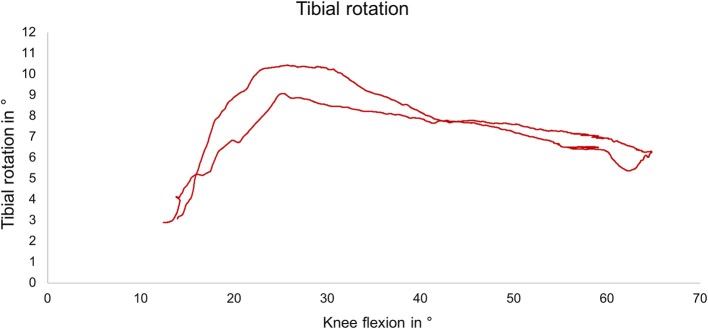
Exemplary external tibial rotation as a function of the knee flexion angle during slow squat with muscle force simulation.

During slow squat movements, a tibial external rotation between ~6° and 12° was determined (Figure 7), reflecting the typical screw home mechanism occurring between knee extension and 30° flexion position.

## Discussion

Within the present study, a novel knee joint simulator was developed and compared to *in vivo* subject data for ground reaction force and muscle forces. It could be shown that this simulator is able to apply rapid movements of 145°/s in combination with physiological muscle force simulation to knee joint specimens that led to realistic ground reaction forces. Therefore, to the best of our knowledge, this knee joint simulator is the first to be able to simulate jump landing movements with physiological joint loads and kinematics.

The proof-of-concept of the knee joint simulator was performed by investigating the ground reaction force in the vertical direction and the tibiofemoral contact pressure. In addition, knee kinematics were analysed to guarantee unconstrained motion.

The ground reaction force is an important measure for the load on the limb (Zadpoor and Nikooyan, 2011). In the present study, it could be shown that during the simulation of a jump landing an almost physiological ground reaction force in the vertical direction could be generated for this cohort with R = 0.93. This physiological ground reaction force during jump landing could be achieved despite partially not perfectly simulated muscle forces. In detail, the muscle force simulation for the quadriceps extensor muscles (M. vastus lateralis, M. vastus medialis, M. vastus intermedius/M. rectus femoris) indicated good correlations (R = 0.72–0.92) between the muscle forces obtained from the MAID study and the simulated muscle forces. In turn, because of the inertia of the pneumatic actuators, the simulation of the hamstring and gastrocnemius muscles was more difficult to achieve. However, the Bravais-Pearson coefficients for these muscle force simulations still showed an acceptable coefficient ranging between R = 0.48–0.71. Additionally, we believe that this did not significantly compromise the resulting knee joint force. These muscle forces act at a much lower force level than, for example, the extensor muscles (Figure 4), and thus contribute less to the stabilisation of the knee joint during jump landing motion than the extensor muscles (Baratta et al., 1988; Urabe et al., 2005). Nevertheless, to improve the simulation of flexor muscle forces in future studies, enhancements in the air pressure control system should be made.

Regarding the tibiofemoral contact pressure, studies have already shown that the contact pressure in the knee joint increased significantly with increasing axial loads even at static knee positions (Poh et al., 2012; Geeslin et al., 2016). Seitz et al. and Perez-Blanca et al. determined peak contact pressures of ~3 MPa while applying an axial load of 1,000 N (Seitz et al., 2012; Perez-Blanca et al., 2016). Lee et al. determined a peak contact pressure of 4.2 MPa in the medial compartment under an axial load of 1,800 N at a knee flexion angle of 60° (Lee et al., 2006). This peak value is slightly lower but at a similar level as the pressure determined in the present study. Therefore, it can be concluded that the axial load generated by the muscle forces during jump landing corresponds to an axial load of ≥1,800 N. It could be further shown, that based on the tibiofemoral contact measurements, a physiological load transfer was achieved only in case of muscle force simulation, indicating a medial to lateral compartment transfer ratio of ~60: 40 (Bruns et al., 1993). In turn, without muscle force simulation the medio-lateral load distribution was random. Comparing the quasi-static like squatting motion without muscle force simulation with the simulated squatting exercise with muscle force simulation and the drop jump led to a significant increase of the tibiofemoral mean and peak contact pressure. This underlines the importance of providing physiological joint forces during *in vitro* experiments.

The analysis of the knee joint kinematics showed an external tibial rotation during simulated slow squatting of ~6° to 12° beginning at 25° of flexion in the present study. This typical screw-home mechanism is an involuntary, passive movement stabilising the knee joint in extension and is caused by the asymmetry between the femoral condyles and the tibial plateau (Piazza and Cavanagh, 2000). According to the literature, the screw-home mechanism begins between 25° and 36° of knee flexion and is normally ~5° to 12° of external rotation (Bull et al., 2008; Müller et al., 2009; Sharma et al., 2012; Hacker et al., 2016). Our measurements are in accordance with these findings, proving the unconstrained motion of the joint specimens.

A limitation of the knee joint simulator introduced here is an observed delay of 120 ms (28%) when comparing the simulation of the jump landing motion (540 ms) and the data obtained from the MAID subject study (420 ms). We assume that the pneumatic actuators were unable to re-adjust rapidly enough, because of the internal pressure control-loop and the inertia of the pneumatic actuators. Nevertheless, with the velocity used for flexion and extension, an almost real-time simulation of a jump landing movement could be achieved. Another limitation is that the MAID study used the Plugin Gait marker set without a complex foot marker set. Since there is not enough resolution in marker-based motion capture to get the precision needed to track the metatarsophalangeal and subtalar joints, especially with only a few marker on the foot, keeping these degrees of freedom locked is well within reason.

In conclusion, the device introduced here can particularly be used for simulating dynamic exercises with rapid movements in combination with physiological muscle forces occurring during daily life. For example, to date, only data about meniscal loads and their attachments from static or quasi-static testing and loading conditions are available. In the future, it will be possible to investigate the loads on the menisci and their attachments under conditions of physiological movements and muscle forces. Other structures, including cruciate and collateral ligaments and cartilage, could also be investigated under such conditions. The knee joint simulator could be extended to include further movement patterns in the future. Consequently, it would be possible to investigate emerging questions, particularly in the field of knee joint trauma and rehabilitation optimisation.

## Data Availability Statement

The datasets generated for this study are available on request to the corresponding author.

## Ethics Statement

The studies involving human participants were reviewed and approved by Ethikkommission Universität Ulm and Ethikkommission Universität Heidelberg. The patients/participants provided their written informed consent to participate in this study.

## Author Contributions

FS developed the knee joint simulator. SD and SW performed the motion analysis and inverse dynamics study. FS and SH performed and evaluated the proof-of-concept tests. FS and AS carried out the statistical analyses. FS, AS, and LD drafted the article and created the figures. AS, AI, and LD participated in the revision process of the article and gave final approval of the submitted version.

### Conflict of Interest

The authors declare that the research was conducted in the absence of any commercial or financial relationships that could be construed as a potential conflict of interest.

## Supplementary Material

Supplementary Video Video of the simulator performing a jump landing exercise.
